# RARβ Agonist Drug (C286) Demonstrates Efficacy in a Pre-clinical Neuropathic Pain Model Restoring Multiple Pathways via DNA Repair Mechanisms

**DOI:** 10.1016/j.isci.2019.09.020

**Published:** 2019-09-17

**Authors:** Maria B. Goncalves, Julien Moehlin, Earl Clarke, John Grist, Carl Hobbs, Antony M. Carr, Julian Jack, Marco Antonio Mendoza-Parra, Jonathan P.T. Corcoran

**Affiliations:** 1 The Wolfson Centre for Age-Related Diseases, King's College London, Guy's Campus, London SE1 1UL, UK; 2 Génomique Métabolique, Genoscope, Institut François Jacob, CEA, CNRS, Univ Evry, Université Paris-Saclay, 91057 Evry, France; 3 Genome Damage and Stability Centre, School of Life Sciences, University of Sussex, Brighton BN1 9RQ, UK

**Keywords:** Biological Sciences, Neuroscience, Transcriptomics

## Abstract

Neuropathic pain (NP) is associated with profound gene expression alterations within the nociceptive system. DNA mechanisms, such as epigenetic remodeling and repair pathways have been implicated in NP. Here we have used a rat model of peripheral nerve injury to study the effect of a recently developed RARβ agonist, C286, currently under clinical research, in NP. A 4-week treatment initiated 2 days after the injury normalized pain sensation. Genome-wide and pathway enrichment analysis showed that multiple mechanisms persistently altered in the spinal cord were restored to preinjury levels by the agonist. Concomitant upregulation of DNA repair proteins, ATM and BRCA1, the latter being required for C286-mediated pain modulation, suggests that early DNA repair may be important to prevent phenotypic epigenetic imprints in NP. Thus, C286 is a promising drug candidate for neuropathic pain and DNA repair mechanisms may be useful therapeutic targets to explore.

## Highlights

• RARβ drug (C286) prevents neuropathic pain after nerve injury
• C286 restores multiple pain pathways to preinjury levels, including MAPK and WNT
• C286 modulates the DNA repair mechanisms BRCA1 and ATM
• BRCA1 is required for C286's effects in neuropathic pain

## Graphical Abstract

**Figure:**
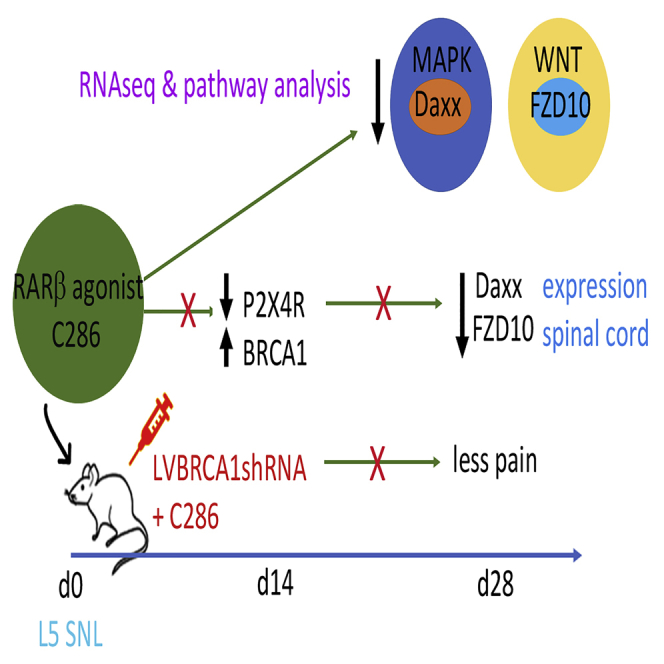

## Introduction

The identification of an effective therapy for neuropathic pain (NP) has been challenging owing to three main factors: first, multiple mechanisms are involved for which no single multifactorial drug has been developed; second, differences in cellular and molecular mechanisms between animals and humans have hampered progress; and third, no single “switch” has been identified that could curtail the pathological cascade and provide a therapeutic target (Borsook et al., 2014, Gereau et al., 2014).

There are two primary features of NP: (1) hyperalgesia, increased pain from a stimulus that usually evokes pain; and (2) allodynia, pain due to a stimulus that usually does not provoke pain (Jensen and Finnerup, 2014). It appears that there are at least two distinct aspects to the development of these features: peripheral sensitization, involving changes in the threshold of peripheral nociceptors including possible spontaneous firing, and central sensitization, in which there are changes in the responsiveness at the central synapses relaying nociception, especially in the dorsal horn of the spinal cord. There is still debate about the importance of central sensitization and whether it relies, for its maintenance, on the peripherally sensitized input (Meacham et al., 2017).

Although it is generally agreed that there are a profusion of gene expression changes in NP (Descalzi et al., 2015), the underlying general mechanism by which they are induced is still uncertain. One suggestion is that the underlying cause is an inflammatory reaction to injury (Ellis and Bennett, 2013), which in turn causes DNA damage (Kelley and Fehrenbacher, 2017). Madabhushi and colleagues (Madabhushi et al., 2015) have shown that even neuronal activity can be sufficient to induce DNA damage (double-strand breaks, DSBs), particularly in the promoter region of early response genes, causing their upregulation, and this, in turn, can alter the expression of late response genes, such as brain-derived neurotrophic factor (*bdnf*). Their experiments supported the conclusion that DNA DSB formation was necessary and sufficient to induce early response gene expression and that DNA repair could reverse the gene expression. Similarly, Fehrenbacher and her colleagues showed that enhanced DNA repair could reverse the changes in neuronal sensitivity that they observed (Kelley and Fehrenbacher, 2017).

In terms of cellular responses, converging lines of evidence support that a specific microglia inflammatory phenotype characterized by the *de novo* expression of the purinergic receptor P2X4 is critical to the induction of core pain signaling, mediated by the release of BDNF, which produces hypersensitivity in nociceptive neuron in the spinal dorsal horn.

It is not understood how this specific spinal microglia phenotype (P2X4R^+^) that arises during the acute stage following peripheral nerve injury (PNI) results in imprinting of the chronic and persistent changes in the spinal nociceptive networks after the acute inflammatory response has subsided (Beggs et al., 2012, Ulmann et al., 2013). Epigenetic alterations in spinal microglia during the acute inflammatory response presents a favorable paradigm for the imprinting mechanism driving chronicity of the pain state (Denk et al., 2016) because of the high transcriptional activity induced by the inflammatory response and the associated increase in DNA DSB (Marnef et al., 2017). Indeed, a wealth of data suggests that the fragility of actively transcribing loci is intertwined with genomic changes that are linked to altered cellular function and disease (Alt and Schwer, 2018, Fong et al., 2013, Puc et al., 2017, Sharma et al., 2015, Su et al., 2015). This raises the question: could this represent a biological switch and thus a therapeutic target, whereby inducing an increase in DNA repair following PNI would preserve the genomic landscape of the spinal microglia during acute activation, when high transcriptional activity is expected, and thus provide an effective way to target NP?

Here we show that a novel drug, Retinoic Acid Receptor (RAR)β agonist, C286 (Goncalves et al., 2019a), prevents NP by restoring pathways that are chronically altered in the spinal cord (SC) after PNI and that this is associated with a switch in the spinal microglia P2X4R phenotype via a mechanism dependent on the breast cancer susceptibility gene 1 (BRCA1). Since the retinoic acid (RA) pathway is highly conserved between species (Rhinn and Dolle, 2012), our findings support C286 as a plausible impending therapy for NP and provide evidence that DNA repair mechanisms are disease-modifying therapeutic targets.

## Results

### C286 Modulates Multiple Pathways Chronically Altered after Spinal Nerve Ligation

RA has been shown to inhibit TNFα and iNOS in reactive microglia (Dheen et al., 2005), and our previous work shows that stimulation of RARβ hampers astrogliosis after spinal cord injury (Goncalves et al., 2015). We therefore hypothesized that a novel drug RARβ agonist, C286, may modulate the inflammatory response of activated microglia to prevent the onset of the microglia-neuron alterations that underly NP. Because we specifically wanted to investigate the effect of the drug in P2X4R^+^ microglia and this phenotype has been shown to evoke spinal mechanisms of nerve injury-induced hypersensitivity predominantly in males but not in female rats (Mapplebeck et al., 2018), we chose male rats only for this study.

**Figure 1:**
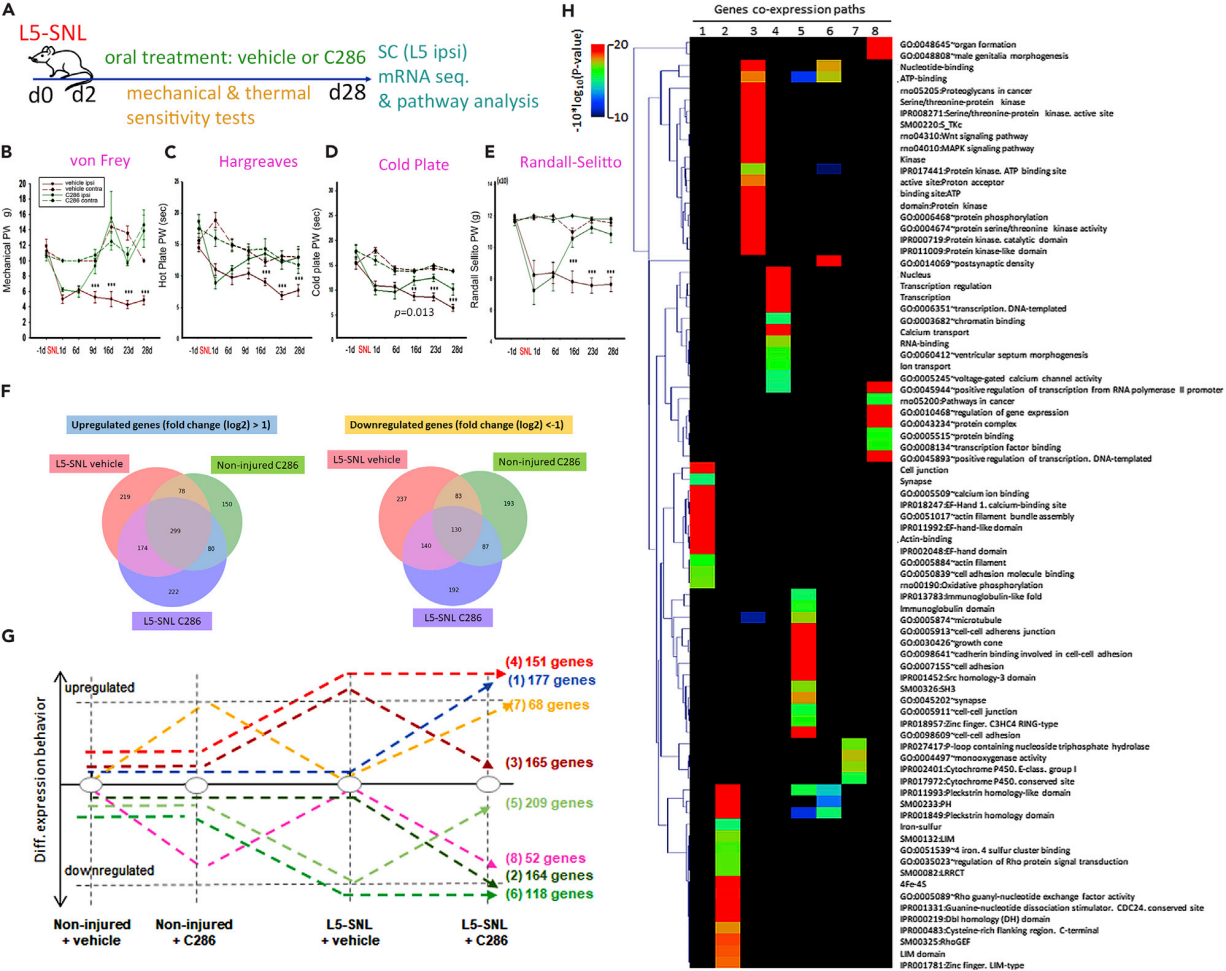
C286 Modulates Multiple Pathways Chronically Altered after SNL (A–E) (A) Schematic of experimental paradigm. L5-SNL, spinal nerve ligation; SC L5 ipsi (spinal cord, L5 level ipsilateral to injury) indicates the area where tissue analysis was carried out and is delineated as a red square in subsequent figures. Measurement of mechanical and thermal sensitivities shown as paw withdrawal (PW) in grams (g) or seconds (sec), by (B) von Frey filaments, (C) hot plate, (D) cold plate, (E) and Randall Selitto test in vehicle (*n* = 8) or C286 (*n* = 8). Data shown as Mean ± SEM. Two-way ANOVA with Pairwise Multiple Comparison Procedures (Holm-Sidak method). **p ≤ 0.01, ***p ≤ 0.001. (F–H) Gene co-expression analysis assessed in the SC from L5-SNL rats and non-injured rats, treated with vehicle or C286. (F) Differential gene expression analysis relative to the sample non-injured + vehicle. (G) Differentially expressed genes are classified by their co-expression paths assessed after injury and injury + vehicle or C286 treatment. (H) Gene ontology analysis performed per co-expression path. The heatmap illustrates the GO enrichment confidence.

**Figure 2:**
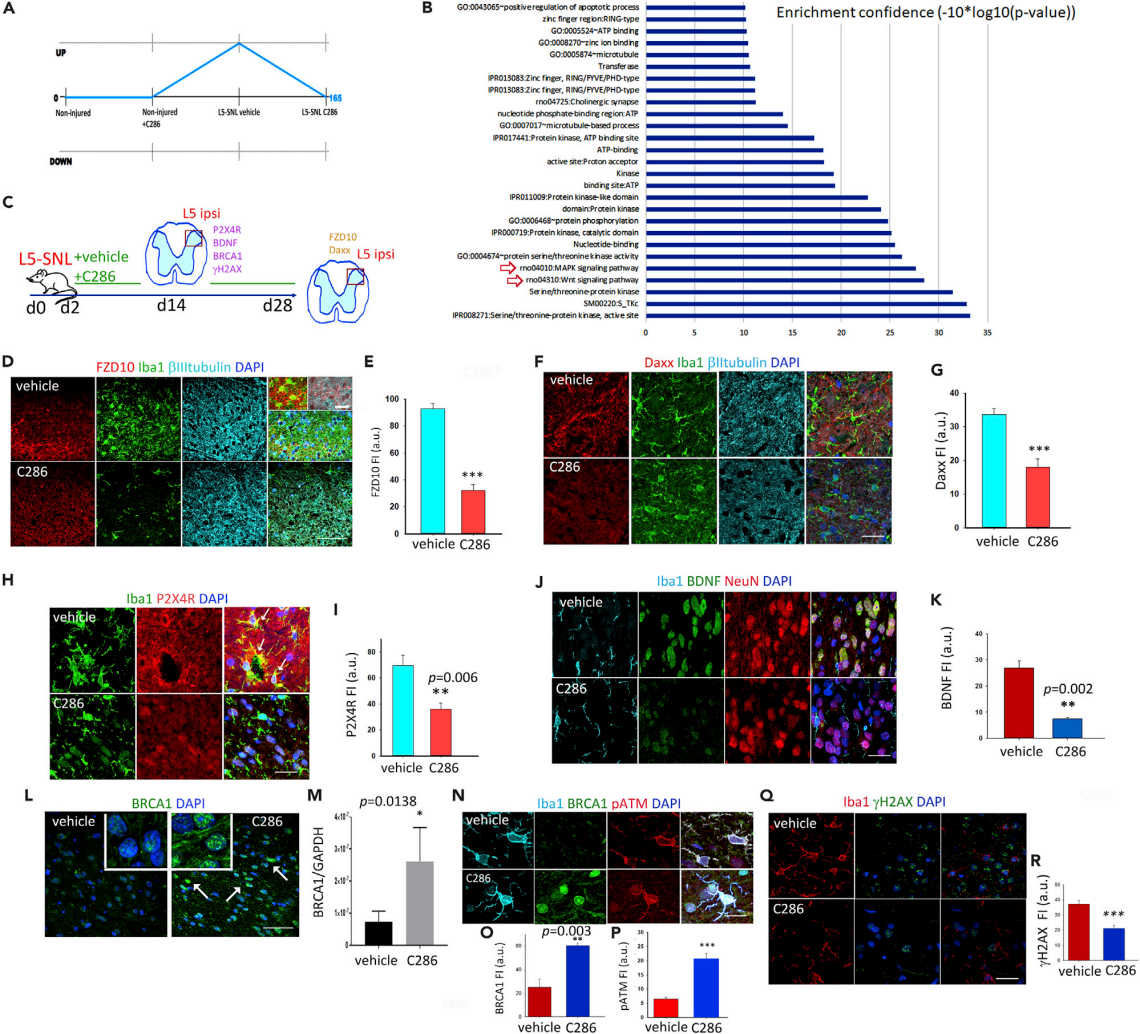
C286 Regulates Inflammatory and DNA Repair Pathways (A) Genes were classified on the basis of their co-expression behavior over the various conditions. C286 downregulated 165 genes that had been upregulated by the injury. (B) GO terms associated to the co-expressed genes are displayed on the basis of their confidence (−10*log10[p value]), red arrows highlight the MAPK and WNT pathways. (C) Diagram of experimental design. (D–G) (D and E) Representative images and quantification of FZD10 (scale bar, 100 μm and 20 μm for higher-magnification insets) and (F and G) of Daxx expression in microglia (Iba1) and neurons (βIII tubulin) in the SC at the end of the treatment period (scale bar, 50 μm). Two weeks after injury, a sub-set of vehicle- and C286-treated rats was used for immunohistological analysis (*n* = 3 per treatment group) and for RT-qPCR (*n* = 3 per treatment group). (H–K) (H and I) Levels of P2X4R^+^ microglia (highlighted by arrows in the merged upper panel, scale bar, 30 μm) and (J and K) BDNF (in neurons and microglia) in the SC (scale bar, 30 μm). (L and M) (L) Images showing BRCA1 expression in the same area (insets show higher magnification of BRCA1 in nuclei, scale bar, 100 μm) and (M) quantification by RT-qPCR. (N–P) Expression and quantification of BRCA1 and pATM in spinal microglia (scale bar, 20 μm). (Q and R) Expression and quantification of γH2AX (scale bar, 100 μm). In E, G, I, K, O, P, and R, data are shown as Mean ± SEM of fluorescence intensity (FI) in arbitrary units (a.u.). Student's t test, **p ≤ 0.01, ***p ≤ 0.001, *n* = 4 (E and G) or *n* = 3 (I, K, M, P, and R) per group, five sections per animal).

Using an established rat model of NP, L5 spinal nerve ligation (SNL) (Kim and Chung, 1992), we assessed the effect of C286 given orally for 4 weeks on mechanical and thermal pain thresholds over the treatment period. C286 treatment reversed the hypersensitivity caused by SNL to levels comparable with the preinjury state (Figures 1A–1E). We next used co-expression analysis of genome-wide RNA sequencing of dorsal horns isolated from non-injured and L5-SNL rats that had been treated with vehicle or C286 to delineate pathways that may have a role in the formation of the long-term hyperalgesia-related imprint in the SC (Figures 1F and 1G). The non-injured tissue was used to establish the normal gene expression with and without C286, whereas the L5-SNL vehicle-treated tissue served as a platform to identify gene expression patterns that were induced by the surgery and peripheral lesion and was used as a control to directly compare gene expression changes that were altered solely owing to the drug treatment. Through analysis of co-expression paths we identified a variety of genes involved in a broad range of cellular functions, including neural transmission, cell adhesion, growth cone and synapse formation, and mitochondrial function (Figure 1H). Among differentially expressed transcripts we identified genes associated with pain-related pathways, altered in different models of pain, or encoding products interacting with proteins involved in pain-related pathways (Figures S1 and S2 and Table S1 [related to Figures 1 and 2]. Figure S2 and Table S1 are available on the Mendeley repository https://doi.org/10.17632/kjvs5vgkbf.1DOI). We observed that C286 upregulates pathways that are compromised in NP: cell adhesion (Patil et al., 2011), growth cone (Hur et al., 2012), and gap junction (Wu et al., 2012) (Figure S2, https://doi.org/10.17632/kjvs5vgkbf.1DOI) and downregulates pathways back to non-injured baseline that are upregulated in NP: long-term potentiation (Ruscheweyh et al., 2011), WNT (Zhang et al., 2013), MAPK (Ji et al., 1999, Jiang et al., 2008, Jin et al., 2003, Kawasaki et al., 2004, Obata and Noguchi, 2004, Song et al., 2005, Zhang and Yang, 2017), erbB (Calvo et al., 2011), TRP channels (Moran and Szallasi, 2018), and cAMP (Edelmayer et al., 2014) (Figures 2A, 2B, and S2 and Table S1, available on the Mendeley repository https://doi.org/10.17632/kjvs5vgkbf.1DOI). Because of their prominent role in the regulation of nociceptive signal perception we focused on the MAPK and WNT pathways for further analysis.

WNT signaling in the SC stimulates the production of proinflammatory cytokines through the activation of WNT/FZ/β-catenin pathway in nociceptive neurons (Tang, 2014, Zhang et al., 2013). MAPK is activated in spinal microglia after PNI and, upon nuclear translocation, activates transcription factors that promote dynamic nuclear remodeling. This results in the transcription and translation of proteins that prolong potentiation and decrease the threshold for receptor activation, the molecular underpinnings of clinical allodynia (Wahezi et al., 2015).

The WNT receptor Frizzled 10 (FZD10) and the death domain-associated protein, Daxx, components of the WNT and MAPK pathways, respectively, were highlighted by our co-expression analysis owing to the magnitude of their expression changes between vehicle and C286-treated L5-SNL rats. FZD10 has been shown to be expressed in pain pathways, including dorsal horn neurons (Hu et al., 2009), and Daxx (which is ubiquitously expressed) has a well-established role in apoptosis but can also participate in numerous additional cellular functions as a mediator of protein interactions (Lindsay et al., 2008), as a potent suppressor of transcription (Takahashi et al., 2004), and as a modulator of cargo-loaded vesicles transport, an important emerging factor in neuron-glia cross-talk during NP (McDonald et al., 2014, Shiue et al., 2019). Immunohistochemistry confirmed downregulation of FZD10 and Daxx protein levels by C286 (see Figures 2C–2G).

### C286 Regulates Inflammatory and DNA Repair Pathways

Inflammation can be a common trigger of MAPK and WNT pathways (Roubert et al., 2017), and hence their downregulation by the agonist at 28 days post injury could indicate an earlier resolution of the inflammatory state. A subtype of purinergic receptor, P2X4R, regulates microglial activation (Ulmann et al., 2013), and its upregulation in spinal microglia has been proposed as an important inflammatory switch that is necessary and sufficient for subsequent pain hypersensitivity, acting via BDNF release and subsequent uptake by the nociceptive neurons in the dorsal horn (Beggs et al., 2012). We therefore assessed the effect of C286 on the expression of P2X4R in spinal microglia and BDNF in microglia and neurons in the dorsal horn at 14 days post PNI, a time point that reflects the interphase between the beneficial acute microglia response and the switch to the perpetuated reactive state that could trigger the chronic pain. We found both to be significantly lower compared with vehicle-treated rats (Figures 2H–2K). Similarly, other inflammatory mediators and growth factors associated with NP, such as NGF, TNFα, and TNFR1 (Amaya et al., 2013), were also downregulated in the agonist-treated SCs (Figures S3A–S3H, related to Figure 2). We did not find their mRNAs upregulated at 4 weeks in the vehicle-treated L5-SNL rats, in agreement with other studies in the same injury model that report only a temporary post-lesion increase in these proteins (de Jager et al., 2011).

Next, we wanted to ascertain if the switch in the microglia phenotype from predominantly P2X4R^+^ to P2X4R^−^ correlated with higher DNA repair efficiency. We reasoned that an increase in DNA repair during the acute phase of microglia activation (Ellis and Bennett, 2013), when transcriptional changes are occurring during adaptation to the injury, could prevent the occurrence of transcriptional imprints that contribute to chronic pain. This would favor regaining the non-activated genomic state. The involvement of the DNA repair protein BRCA1 in spinal microglia after injury has been recently described where an initial physiological attempt to repair is seen by an increase in BRCA1 expression, but that is not sustained beyond 72 h post injury (Noristani et al., 2017). A link between BRCA1 and RA signaling has been highlighted by previous studies; genome-wide analysis suggests a role for BRCA1 in transcriptional co-activation to RA (Gardini et al., 2014) and RAR/RXR-mediated transcription requires recruitment of the BRCA1 co-repressor C-terminal-binding protein 2 (CtBP2) (Bajpe et al., 2013), which could result in the elevation of BRCA1 transcription, a mechanism already described for estrogen (Di et al., 2010).

To assess if C286 could be prolonging BRCA1 expression, we measured BRCA1 levels in the dorsal horn by western blotting (Figures 2L and 2M) and by immunochemistry in spinal microglia and found that C286 significantly increased BRCA1 levels, predominantly in the nucleus (Figures 2L, 2N, and 2O).

### C286 Regulates DNA Damage in Microglia via BRCA1 and ATM Pathways

**Figure 3:**
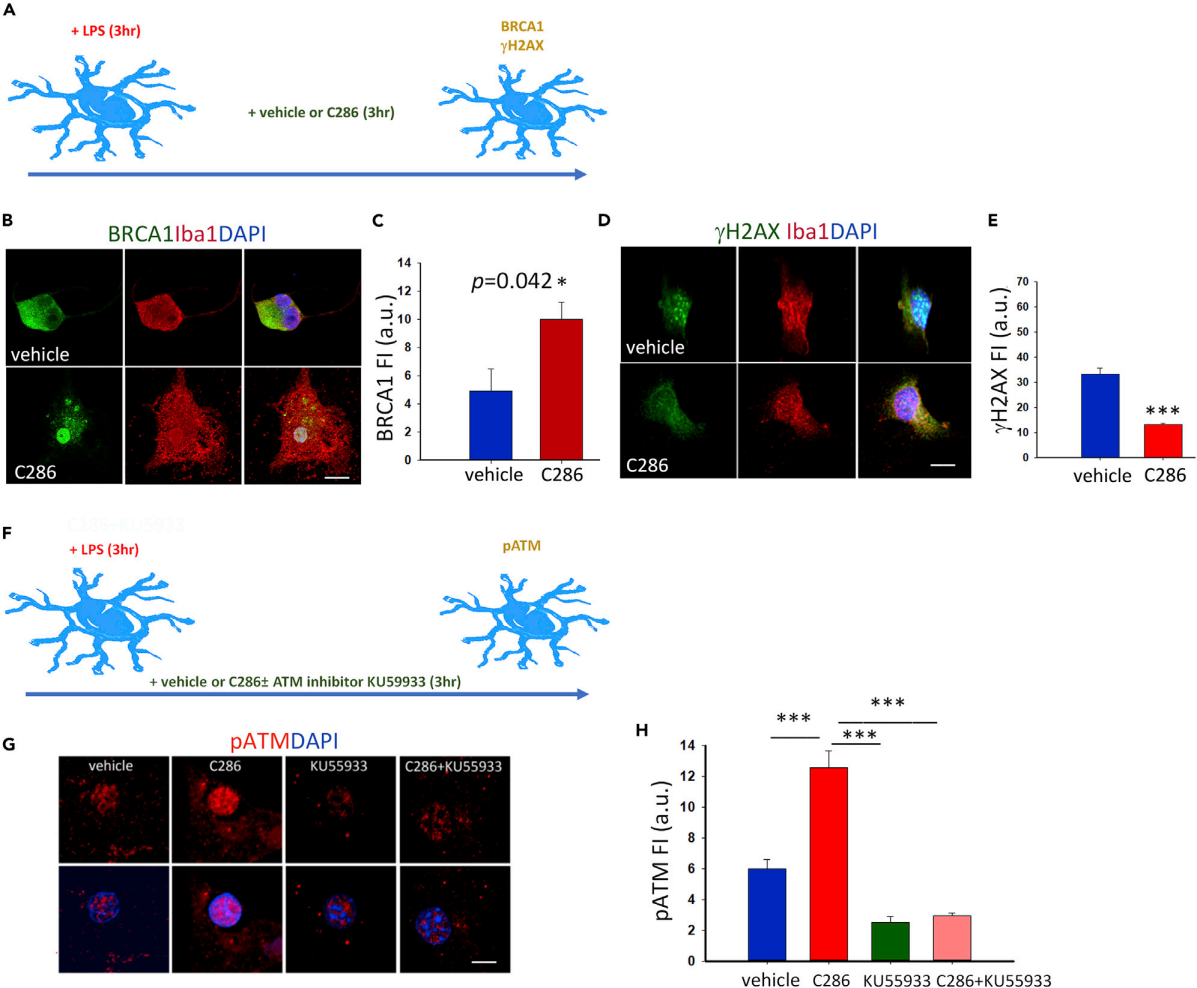
C286 Regulates DNA Damage in Microglia via BRCA1 and ATM Pathways (A) Diagram showing microglia culture conditions and markers assessed. (B–E) (B and C) BRCA1 and (D and E) γH2AX expression and quantification in microglia cultures. (F) Diagram showing the experimental design. (G and H) Expression and quantification of pATM in nuclei in the different culture conditions. Scale bars, 15 μm. Data show Mean FI ± SEM from three independent experiments (C and E), Student's t test, *p ≤ 0.05 and (H) one-way ANOVA with Pairwise Multiple Comparison Procedures (Tukey Test), ***p ≤ 0.001.

Cellular responses to DNA damage are mediated by an extensive network of signaling pathways. The ataxia telangiectasia mutated (ATM) kinase responds specifically to DNA DSBs, which are associated with signal-induced transcriptional changes. ATM can be activated by RA (Fernandes et al., 2007) and suppresses MAPK pathways via a DSB-induced response whereby MKP-5 is upregulated and dephosphorylates and inactivates the stress-activated MAP kinases JNK and p38 (Bar-Shira et al., 2002). We therefore assessed ATM phosphorylation levels in the SCs and found that C286 significantly increased pATM in spinal microglia (Figures 2N and 2P). Concomitantly, we observed a significant decrease in the ATM target and DNA damage marker γH2AX (Sharma et al., 2012) (Figures 2Q and 2R). To confirm if the modulation of these two DNA repair mechanisms was a direct effect of the agonist in microglia, we treated lipopolysaccharide-activated microglia cultures with vehicle, C286, an ATM inhibitor (KU55933) alone, or with C286 and found that C286 significantly increased BRCA1 and pATM and significantly decreased γH2AX compared with vehicle. Importantly, the effect on pATM was completely abrogated in the presence of KU55933, suggesting a direct effect on ATM auto-phosphorylation (Figures 3A–3H).

### BRCA1 Is a Downstream Target of C286 in NP Modulation

**Figure 4:**
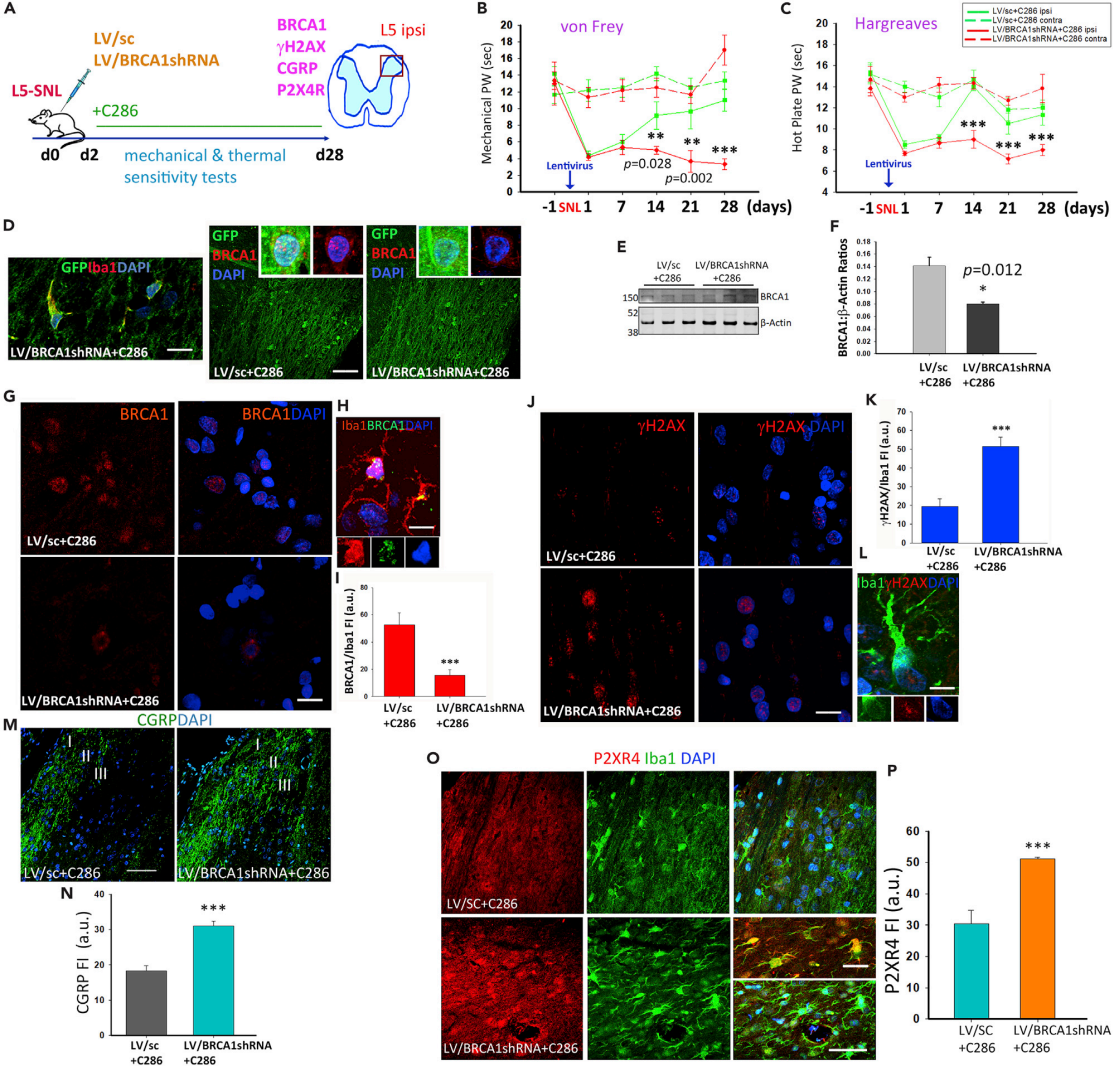
BRCA1 Is a Downstream Target of C286 in NP Modulation (A) Schematic of experimental paradigm. (B and C) (B) Measurement of thermal and mechanical sensitivities by von Frey filaments (C) and hot plate in vehicle (*n* = 6) or C286 (*n* = 6). Data shown as Mean ± SEM. Two-way ANOVA with Pairwise Multiple Comparison Procedures (Holm-Sidak method). ***p ≤ 0.001. (D) Co-labelling of GFP and Iba1 (scale bar, 20 μm), and GFP and BRCA1 in LVsc + C286- and LVBRCA1shRNA + C286-treated rats (scale bar, 100 μm). (E–H) (E and F) Western blots and quantification of BRCA1 in L5-SNL rats transduced with LV/SC + C286 (*n* = 3) or LV/BRCA1shRNA + C286 (*n* = 3). Student's t test. *p ≤ 0.05 (G) Immunohistological confirmation of LV transduction (scale bar, 20 μm), (H) higher-magnification inset shows BRCA1 in microglia in an LV/Sc + C286-treated rat (scale bar, 10 μm). (I) Quantification of BRCA1 in microglia. (J and K) Expression and quantification of γH2AX (scale bar, 20 μm), (L) higher-magnification inset shows γH2AX in microglia in an LV/BRCA1shRNA + C286-treated rat (scale bar, 10 μm). (M and N) Expression and quantification of CGRP in laminae I–III of the dorsal horn (scale bar, 100 μm). (O and P) Expression and quantification of P2X4R (scale bar, 100 μm). Inset shows higher-magnification image (scale bar, 20 μm) Data are shown as Mean +SEM of FI. Student's t test, **p ≤ 0.01, ***p ≤ 0.001 (*n* = 3 per group, 5 sections per animal).

To functionally validate the RARβ-BRCA1 pathway in pain we used lentiviral transduction of shRNA BRCA1 in our rat model of NP. Treatment with C286 yielded no significant improvement in the pain thresholds when BRCA1 was ablated (Figures 4A–4C). Confirmation of effective lentiviral transduction was obtained by immunochemistry (Figures 4D–4F). Further analysis of BRCA1 expression in spinal microglia showed that this was significantly decreased in LV/BRCA1shRNA + C286-treated rats compared with LV/sc + C286 (Figures 4G–4I), and the inverse was seen with γH2AX (Figures 4J–4L). In agreement with the pain behavioral tests, we found that the calcitonin gene-related peptide (CGRP), which contributes to the hypersensitization (Iyengar et al., 2017), was significantly upregulated in the dorsal horn (predominantly laminae I-III) of LV/BRCA1shRNA + C286-treated rats (Figures 4M and 4N).

**Figure 5:**
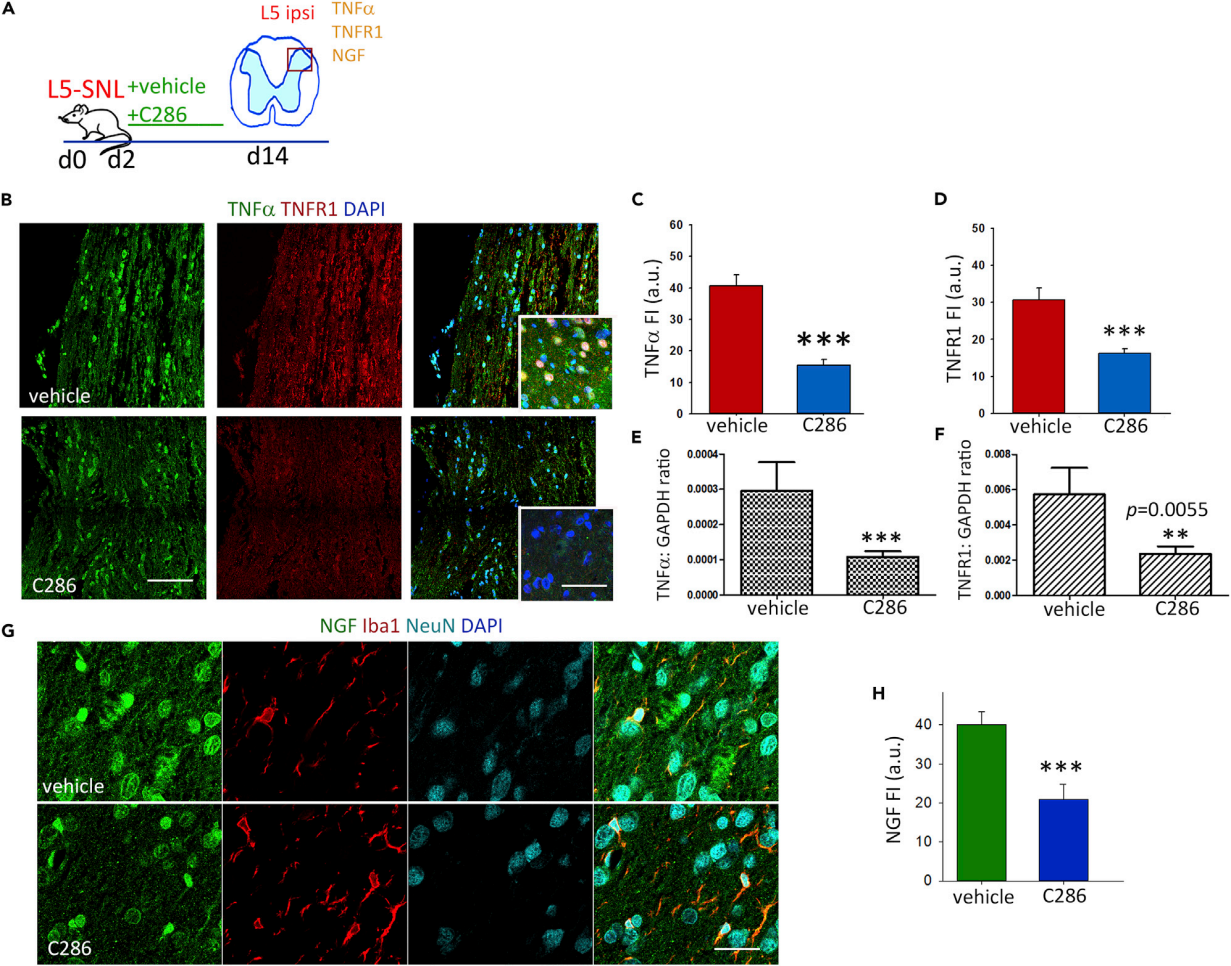
BRCA1 Influences Inflammatory Mechanisms in NP (A) Diagram of the experimental design. (B–G) (B) Immunostaining of spinal microglia and NGF, (D) TNFα, and (F) TNFR1 in LV/sc + C286- and LV/BRCA1shRNA + C286treated rats (scale bars, 30 μm). Quantification of FI for (C) NGF, (E) TNFα, and (G) TNFR1. Data show Mean FI ± SEM, Student's t test, ***p ≤ 0.001 (*n* = 3 per group, 5 sections per animal).

**Figure 6:**
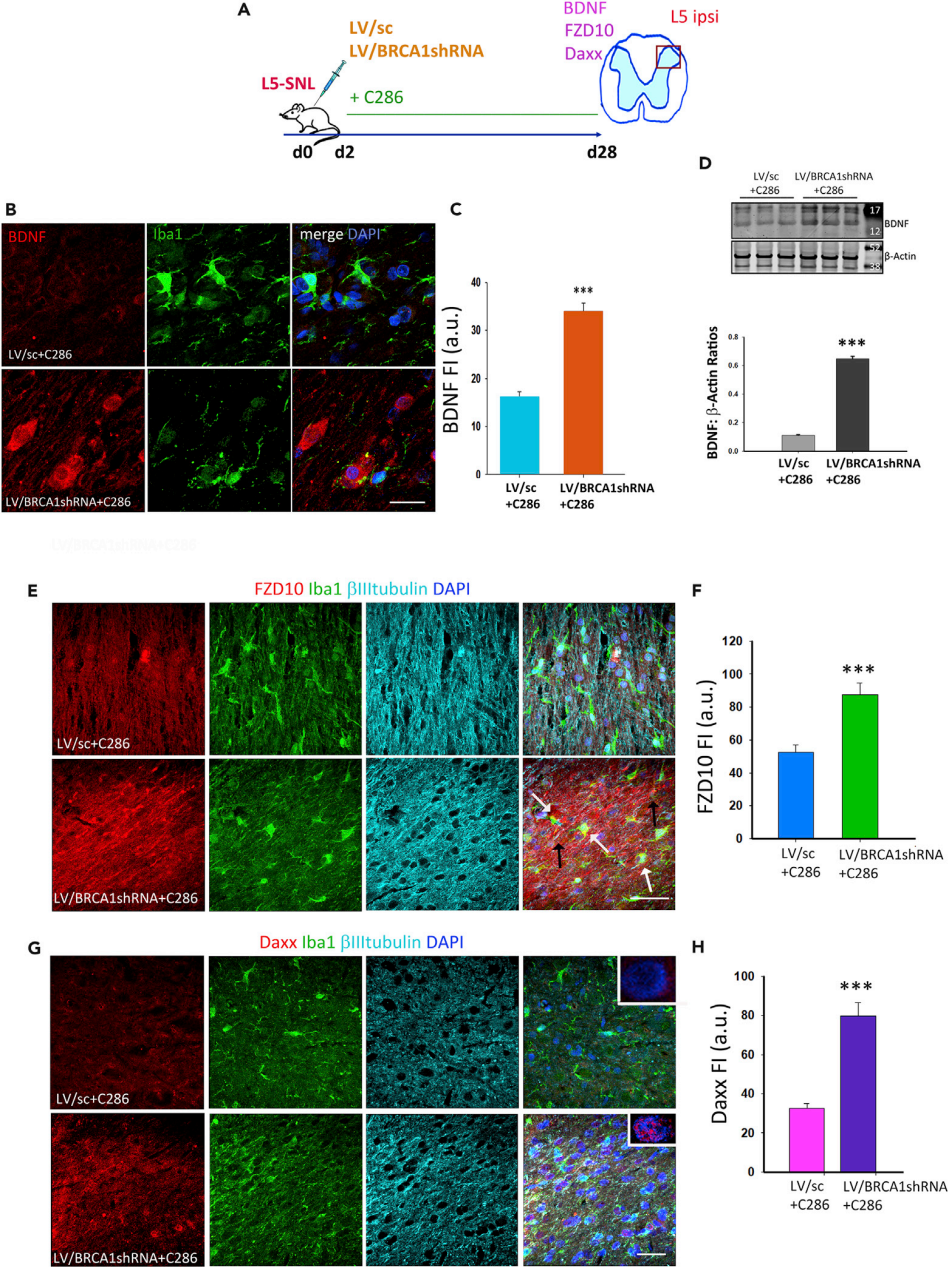
BRCA1 Is Necessary for C286-Mediated Regulation of Pain (A) Diagram of the experimental design. (B–D) (B and C) Immunostaining of spinal microglia for BDNF (scale bar, 50 μm) and its quantification. (D) Western blots of spinal cords for BDNF. Student's t test, n = 3 per group, ***p ≤ 0.001. (E–H) (E and F) Expression and quantification of FZD10 (white arrows show colocalization with Iba1 and black arrows with βIII tubulin) and (G and H) Daxx in spinal neurons and microglia of LV/sc + C286- and LV/BRCA1shRNA + C286-treated rats (insets show its predominant nuclear localization). Scale bars, 100 μm for E and G. Data show Mean FI ± SEM, *n* = 3 per treatment group, 5 sections per animal. Student's t -test, **p ≤ 0.01, ***p ≤ 0.001.

To establish if there was a direct link between BRCA1 and the microglia activation, we assessed the levels of P2X4R in spinal microglia and found a significant increase in the LV/BRCA1shRNA + C286-transduced rats (Figures 4O and 4P). This effect was also seen for NGF, TNFα, and TNFR1 (Figures 5A–5G), indicating that an inflammatory environment was still present in the SC. Concurrent protein expression analysis of BDNF and the components of the MAPK and WNT pathways (FZD10 and Daxx), which had been modified by the agonist in L5-SNL non-transduced rats (see Figure 2), showed a significant increase with the suppression of BRCA1 despite the agonist treatment (Figures 6A–6H).

## Discussion

Collectively, we show that C286 generates a “repair proficient” environment that may influence epigenetic modification of some enhancers in microglia, resetting the transcriptome toward a resting state after injury and thus reducing the long-term transcription of NP-associated genes. C286 modulates DNA repair mechanisms involving BRCA1 and ATM in spinal microglia, the former being directly linked to the P2XR4 phenotype and the development of NP. This supports the concept that transcription-induced persistent damage that is inefficiently repaired could chronically alter the epigenetic landscape, in line with the emerging importance of BRCA1 in neurodegenerative diseases (Mano et al., 2017, Suberbielle et al., 2015). Current therapeutic strategies generally aim at a single molecular target. These are yielding unsatisfactory results and are thus giving ground to a multifactorial approach targeting the numerous pathways involved, one possibility being to influence DNA repair mechanisms (Kelley and Fehrenbacher, 2017). Here we show that C286 has multiple effects on pathways that contribute to the chronicity of the neuronal sensitivity and thus might prove a more successful approach for the treatment of NP.

### RARβ Signaling and WNT/FZD Signaling

We found that the WNT pathway is one of the most significantly downregulated pathways by the agonist. The importance of the WNT/FZD signaling in signal transduction and synaptic plasticity alterations, which are essential to SC central sensitization after nerve injury, has been documented before (Zhang et al., 2013, Zhao and Yang, 2018). It is thought that WNT/FZD/β-catenin signaling contributes to the onset and persistence of pain after nerve injury, through activation of signaling pathways that recapitulate development, such as axon guidance, synaptic connection, and plasticity in the spinal cord. Spinal blockade of WNT signaling can inhibit the production and persistence of PNI-induced NP and prevent upregulation of the NR2B receptor and the subsequent Ca^2+^-dependent signals CaMKII, Src/Tyr418, pPKCγ, ERK, and cAMP response element-binding protein within the SC pain pathways (Zhang et al., 2013). Curiously, we found that C286 suppresses WNT/FZD signaling and upregulates pathways involved in regeneration, which are also important during development. This may seem an incongruence, but we must consider that the overall biological effect is determined by a network of interacting pathways. WNT is known to interact with ephrinB-EphB receptor signaling, which also activates various developmental processes of the nervous system in response to nerve injury (Han et al., 2008, Song et al., 2008) and is thought to contribute to pain enhancement. These interactions may result in an exacerbation of neurochemical signs within development pathways that trigger and sustain pain pathways. Therefore, it is likely that the C286-mediated stimulation of the regeneration and development pathways is quite different, both qualitatively and quantitatively, because C286 upregulates transcription of these pathways to preinjury levels but not beyond. This promotes the restoration of homeostasis and prevents activation of pathways that sustain pain.

### RARβ and MAPK/Daxx Signaling

It is interesting that Daxx showed the highest downregulation within the MAPK pathway. Daxx is associated mostly with triggering apoptotic pathways that result in cell death and/or senescence. The agonist prevented the upregulation of Daxx in response to the injury and concomitantly upregulated various other pathways that are associated with normal cellular functions: cell-adhesion, mitochondria function, etc. The counterpart scenario, i.e., the downregulation of these pathways in the vehicle-treated rats, possibly reflects a state of compromised cellular functions in the SC. Therefore, it seems that Daxx could be an important contributor to cell fate in PNI-induced NP in the SC.

We found that RARβ activation downregulates TNF-α, which is one of the cytokines that induces phosphorylation and stabilization of Daxx through ASK1 activation. This is essential for activation of the pain signaling pathways, JNLK and p38 (Chang et al., 1998, Ichijo et al., 1997, Ji et al., 2009). Thus, it is possible that the marked downregulation of Daxx by C286 is in part a consequence of the agonist's anti-inflammatory effect. Similarly, the prevention of the reactive microglia P2X4R phenotype could be a direct consequence of a milder inflammatory milieu facilitated by the acute agonist action. Nonetheless, the overall effect of C286 cannot be justified entirely and solely by an initial anti-inflammatory effect. If that was the case, then anti-inflammatory treatment would be a successful therapeutic approach. Arguably, it is a combination of different mechanisms directly and indirectly affecting various intracellular functions: DNA repair, transcription, organelle transport, energy supply, and secretion of signaling molecules, which contributes to the RARβ modulation of NP.

### RARβ and the Extracellular Matrix

C286 also induced an upregulation of cell adhesion and cell junction pathways. This is noteworthy because adhesion proteins, which normally build and modify synapses, also participate in different aspects of synaptic and circuit reorganization associated with NP (Dina et al., 2004).

### C286 as a Promising Transcriptional Drug

We challenge the dogma that nuclear receptor agonists are unpromising therapeutic targets. Nuclear receptor signaling has been overlooked as a therapeutic avenue. Although nuclear receptor signaling regulates many pathways, it is thought that some of these might be detrimental to the cells casting doubt on the overall biological effect. However, effective therapies need to be multifactorial, especially if they are aimed at chronic conditions in which a myriad of cellular functions has been altered. Retinoic acid modulates transcription and exerts its biological activity via the nuclear receptor RAR/RXR heterodimers, of which three isoforms have been identified (α,β,γ) (reviewed in Maden, 2007). Each isoform differs in spatial expression and yields different biological responses. In this regard, it is therefore beneficial to use specific receptor agonists targeted to the particular receptor that will induce the desired/anticipated effect. Because RXRs are promiscuous receptors and partner with various other nuclear receptors integrating their signaling pathways (Lefebvre et al., 2010), they are less attractive as drug targets. We have demonstrated target engagement previously and shown the upregulation of RARβ in response to treatment with specific RARβ agonists (Goncalves et al., 2018, Goncalves et al., 2019b). Our work illustrates an example of where a nuclear receptor agonist provides an effective treatment for a chronic condition without induction of detrimental pathways. C286 is currently undergoing a phase 1 trial (ISRCTN12424734) and can rapidly progress to further clinical testing proving an attractive therapeutic avenue to explore for NP.

### DNA Damage Pathways and Future Therapeutic Avenues

DNA damage has recently been proposed to play an important role in transcriptional regulation. Here we show that it is involved in setting an inflammatory state in spinal microglia that triggers NP. Our results demonstrate a novel role for BRCA1 in NP. BRCA1 is a DNA repair protein, best known for its association with breast cancer. We demonstrate that, by increasing DNA repair via BRCA1, NP can be prevented. This revolutionizes the therapeutic exploration for NP, shifting its focus from targets whose modification provides symptomatic and temporary amelioration to a more permanent disease-modifying target: DNA repair. Recovery of normal cellular functions through effective and timely DNA repair might be a successful prophylactic and/or therapeutic approach that is extendable to other chronic conditions similarly associated with an inflammatory etiology. Exploring other drugs that, like C286, modulate BRCA1 and identifying other key DNA repair mechanisms could be a step change in therapeutic development.

### Limitations of the Study

This study was conducted in male rats only and as such does not address the sexual dimorphism in pain.

## Methods

All methods can be found in the accompanying Transparent Methods supplemental file.

## Supplemental Information

Document S1. Transparent Methods and Figures S1–S3

Table S1. Summary of the GO Terms for All 20 Co-expression Paths, Related to Figures 1 and 2 (https://doi.org/10.17632/kjvs5vgkbf.1DOI) The p-value is expressed in "-10*log10(p-value)" and for each path it is indicated their corresponding status (i.e., [0][1][0] indicated the expression status for [Non-Injured+C286][Injured+Veh][Injured+C286]).
