# Supplementary material for: RARβ Agonist Drug (C286) Demonstrates Efficacy in a Pre-clinical Neuropathic Pain Model Restoring Multiple Pathways via DNA Repair Mechanisms
Source: iScience. 2019 Sep 17;20:554–66. doi: 10.1016/j.isci.2019.09.020 (PMC6833472; doi:10.1016/j.isci.2019.09.020)
Supplement: Document S1. Transparent Methods and Figures S1–S3 [file mmc1.pdf]

ISCI, Volume *20*

Supplemental Information

RARβ Agonist Drug (C286) Demonstrates Efficacy in a Pre-clinical Neuropathic Pain Model Restoring Multiple Pathways via DNA Repair Mechanisms

Maria B. Goncalves, Julien Moehlin, Earl Clarke, John Grist, Carl Hobbs, Antony M. Carr, Julian Jack, Marco Antonio Mendoza-Parra, and Jonathan P.T. Corcoran

A

	Number of reads before alignment	Number of reads after alignment	Percentage of reads aligned
JC12	24 512 008	20 107 198	82.03%
JC13	27 181 807	21 410 429	79.00%
JC16	37 095 298	29 672 629	79.99%
JC17	29 078 945	22 611 798	77.76%
JC18	23 885 123	19 134 372	80.11%
JC48	23 770 229	19 318 064	81.27%
JC49	26 164 034	21 158 258	80.86%
JC50	24 078 835	19 519 000	81.07%
JC51	27 756 882	22 773 701	82.05%
JC52	27 405 453	22 280 301	81.21%
JC53	25 408 422	20 682 877	81.17%

B

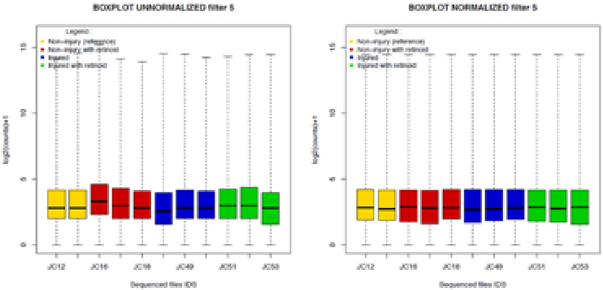

C

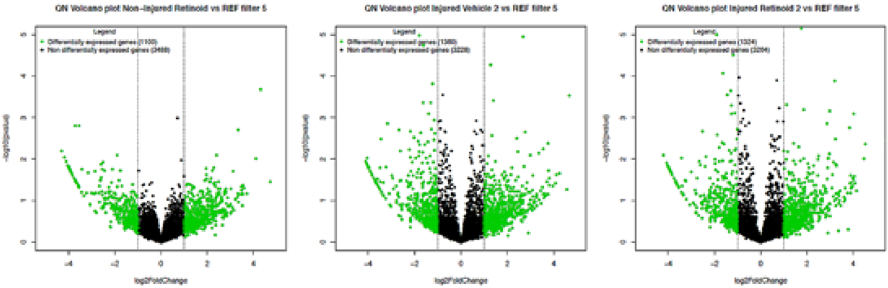

Co-expresion path 1

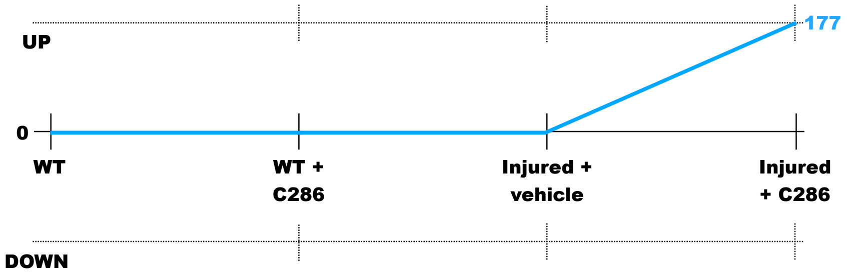

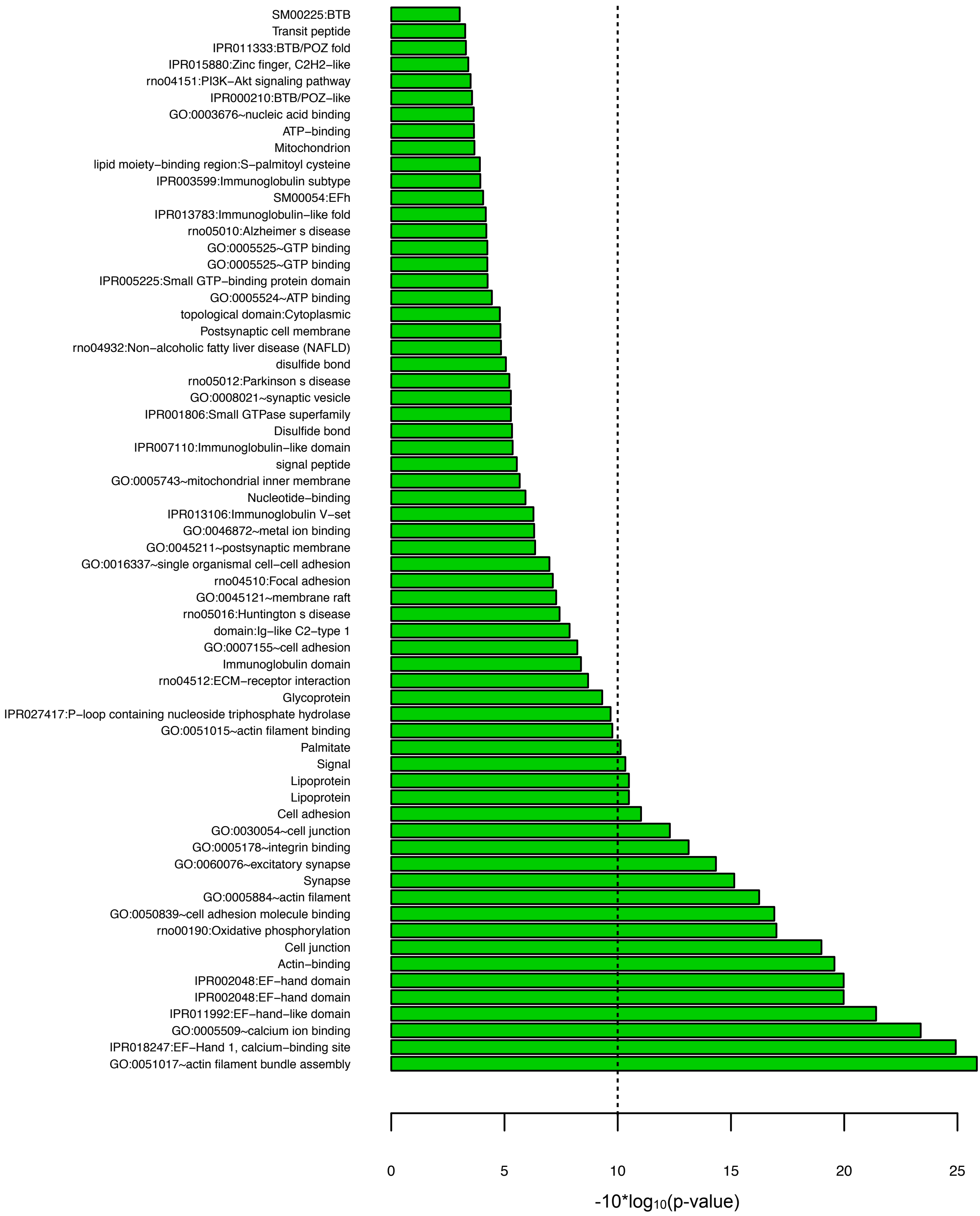

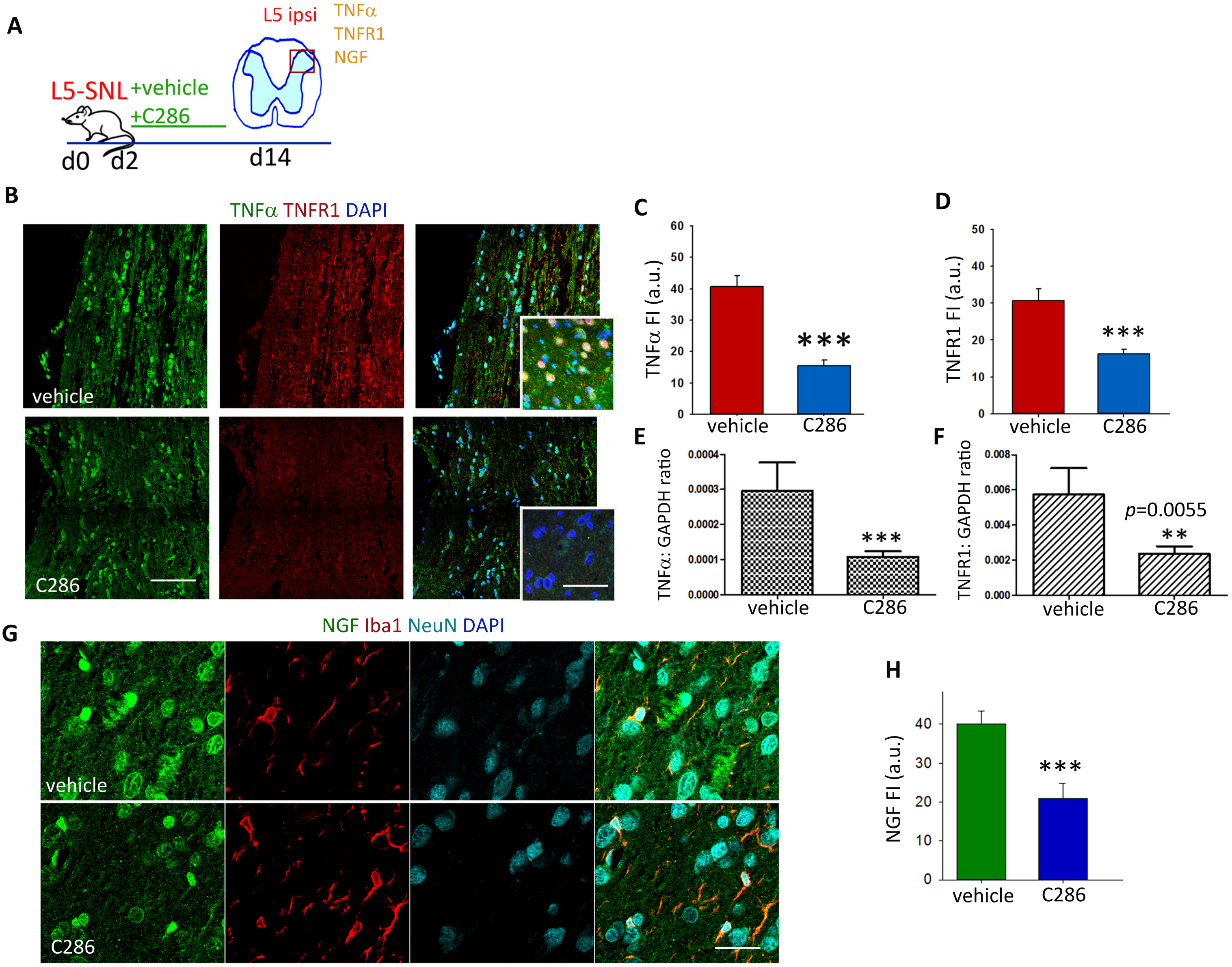
A
L5 ipsi
TNFα
TNFR1
NGF
L5-SNL
+vehicle
+C286
d0
d2
d14
B
TNFα TNFR1 DAPI
vehicle
C286
C
TNFα FI (a.u.)
vehicle
C286
D
TNFR1 FI (a.u.)
E
TNFα: GAPDH ratio
F
TNFR1: GAPDH ratio
p=0.0055
G
NGF Iba1 NeuN DAPI
H
NGF FI (a.u.)

Figure S1. Transcriptomics analysis of spinal cord samples issued from animals under different conditions

A, Summary of the number of DNA sequenced reads obtained per sample as well as the fraction retained after alignment to the Rattus norvegicus reference Genome. **B,** Boxplots depicting the read counts per gene prior or after quantile normalization. **C**, Volcano plot illustrating the fraction of differentially expressed genes (rel. to non-injured + vehicle condition), as defined by a two-fold change criterion, and their related confidence (p-value).

Figure S2. Gene Ontology (GO) enrichment analysis on co-expressed genes (http://dx.doi.org/10.17632/kjvs5vgkbf.1DOI)

Considering 3 differentially expressed conditions (non-injured + C286; injured + vehicle; injured + C286) and three gene expression states (induced, repressed and non-differentially expressed), a total of 26 combinatorial gene co-expression events are theoretically possible. Genes were classified on the basis of their co-expression behavior over the various conditions**.** GO terms associated to the co-expressed genes are displayed on the basis of their confidence (-10*$\log_{10}$(p-value), as inferred by the DAVID bioinformatics resources (Huang da et al., 2009). Co-expression path 1 is shown here, all the other paths are available in **(http://dx.doi.org/10.17632/kjvs5vgkbf.1DOI).**

Figure S3. Modulation of TNFα, TNFR1 and NGF by C286

a, Diagram of the experimental design. **b-d,** Immunostaining and quantification of TNFα and TNFR1 by IF (scale bars are 100μm and 50 μm for insets) and by **e,f,** RT-qPCR. Student's *t*-test, $**p \leq 0.01$, $***p \leq 0.001$, (n= 3 per group). **g,** NGF expression in spinal neurons and microglia (scale bar is 30μm) and **h**, quantification of total FI. Data shows Mean FI ± SEM. Student's *t*-test, $**p \leq 0.01$, $***p \leq 0.001$, (n= 3 per group, 5 sections per animal).

Table S1. Summary of the GO terms for all 20 co-expression paths (http://dx.doi.org/10.17632/kjvs5vgkbf.1DOI)

The p-value is expressed in "-10*log10(p-value)" and for each path it is indicated their corresponding status (i.e. [0][1][0] indicated the expression status for [Non-Injured+C286][Injured+Veh][Injured+C286]).

Transparent Methods

Rats and animal procedures

All procedures were in accordance with the UK Home Office guidelines and Animals (Scientific Procedures) Act of 1986. Male Sprague Dawleys rats, weighing 220-250 grams were used throughout the study. All animal care and experimental procedures complied with the Animals (Scientific Procedures) Act, 1986 of the UK Parliament, Directive 2010/63/EU of the European Parliament and the Guide for the Care and Use of Laboratory Animals published by the US National Institutes of Health (NIH Publication No. 85–23, revised 1996). Animal studies are reported in compliance with the ARRIVE guidelines (Kilkenny et al., 2010; McGrath and Lilley, 2015). All surgery, behavioural testing and analyses were performed using a randomized block design and in a blinded fashion. Allocation concealment was performed by having the treatment stocks coded by a person independent of the study. Codes were only broken after the end of the study.

Animals were housed in groups of three to four in Plexiglas cages with tunnels and bedding, on a 12:12 h light/dark cycle and had access to food and water ad libitum. Experimental neuropathy was induced by an L5 spinal nerve ligation (Bennett et al., 2003) (L5 SNL-2 week analysis; *n*= 6 per treatment group; L5 SNL- 4 week analysis *n*= 8 per treatment group; L5 SNL with lentivirus; n=6 per treatment group)**.** Briefly, rats were anesthetized via intraperitoneal injection of 0.25 mg kg^{-1} medetomidine/60 mg kg^{-1} ketamine solution, following which the vertebral transverse processes were exposed via a small skin incision and

retraction of the paravertebral musculature. The L6 transverse process was partially removed and the L5 spinal nerve was identified, tightly ligated, and sectioned 1–2 mm distal to the silk ligature. During the surgery, the rats were placed on a controlled heating pad to maintain temperature at 37 ± 1°C. After the surgery, the rats were hydrated with physiological saline (2 mL, s.c.). Anesthesia was reversed with an intra muscular (IM) injection of 0.05 ml (1 mg/kg) atipamezole hydrochloride (Antisedan®; Pfizer Animal Health, Exton, PA). Animals were kept in a heated recovery box until fully conscious and analgesia (buprenorphine, 0.01 mg/ kg, subcutaneously) was given after suturing and recovery. All the rats survived this surgery.

For BRCA1 loss of function studies, at the time of the L5 SNL, 5 μl of lentivirus (titer: 3.67×10^8 TU/ml), either Brca1 Rat shRNA Lentiviral Particle, sequence, TACACAGCCTGGTGTCTCTAAGCAGAGTG, or scrambled, sequence, GCACTACCAGAGCTAACTCAGATAGTACT (TL709162V, Origene**)** was injected manually into the spinal cord at the level of L5, using a 20 μl Hamilton syringe at 0.5 μl min^{-1} and the needle was left in place for the following 5 minutes to limit diffusion through the needle tract.

Drug treatments and tissue processing for in vivo studies

Rats were treated with vehicle or a novel selective RARβ agonist, C286 (Goncalves et al., 2019a) (synthesized by Sygnature Chemical Services, Nottingham, UK), given by oral gavage (po) three times a week for four weeks at 3mg/kg. C286 has a high potency at RARβ (similar potency to all-trans-retinoic acid) and behaves as a full agonist showing an EC_{50} of 1.94 nM at the mouse RARβ receptor and a selectivity for RARβ over RARα of 13.4, with selectivity for RARβ over RARγ being 5.6-fold (Goncalves et al., 2019a).

After defined survival times, animals were killed by terminal anesthetization and transcardially perfused with 4% paraformaldehyde, for immunohistochemistry or ice-cold PBS, for mRNA

extraction and Western blotting. The lumbar spinal cords were excised, rapidly removed and the tissue corresponding to L5 spinal cord was isolated. For immunohistochemistry, the tissue was post-fixed with 4% PFA for at least two days at room temperature before being embedded in paraffin wax. Five μm longitudinal or transverse sections cut throughout each block. Sets of consecutive sections, comprising the L5 lumbar spinal cord were taken and used for immunohistochemical analysis. For mRNA extraction and Western blotting, L5 spinal cords were bi-dissected into ipsi and contralateral side to the injury, and tissue was snap frozen in liquid nitrogen and kept at -80 ^{0}C until further use.

Behavioural tests

Pain thresholds were measured according to the methods described below. Baseline recordings were taken at 1 day prior to surgery and at 1, 6, 9, 16, 23 and 28 days after surgery for L5 SNL experiment and at 1, 7, 14, 21 and 28 days for the L5 SNL lentivirus study.

Mechanical thresholds

von Frey test

Static mechanical withdrawal thresholds were assessed by applying von Frey hairs (Touch Test, Stoelting, IL, USA) to the plantar surface of the hind paw. Unrestrained animals were acclimatised in acrylic cubicles (8 x 5x 10 cm) on a wire mesh grid for 60 min prior to testing. Calibrated von Frey hairs (flexible nylon fibres of increasing diameter that exert defined levels of force) were applied to the plantar surface of the hind paw until the fibre bent. The hair was held in place for 3 secs or until the paw was withdrawn in a reflex not associated with movement or grooming. Each hair was applied 5 times to both the left and the right paw alternately, starting with the lowest force hair. Hairs of increasing force were applied in

sequence, 5 applications per hair. A positive response was recorded when a 40% withdrawal response occurred over 5 applications.

Randall-Selitto test

Mechanical nociceptive thresholds were evaluated using an Analgesy-Meter (Ugo Basile, Comerio, Italy). Rats were gently held and incremental pressure (maximum 25 g) was applied onto the dorsal surface of the hind paw. The pressure required to elicit paw withdrawal, the paw pressure threshold, in g was determined. An average of three recordings were taken separated by at least 5 minutes.

Thermal thresholds

Hot plate- Hargreaves test

Briefly, the rats were habituated for 10 minutes to an apparatus consisting of individual Plexiglas boxes on an elevated glass table. A mobile radiant heat source was located under the table and focused on the hind paw, and the paw withdrawal latencies were defined as the time taken by the rat to remove its hind paw from the heat source. The cut-off point was set at 25 s to prevent tissue damage. The apparatus was calibrated to give a paw withdrawal latency of approximately 15 s in naïve rats. An average of three recordings were taken separated by at least 5 minutes.

Cold plate

Withdrawal of the hind paw in response to painful cold stimulation was assessed using an Incremental Hot/Cold Plate (IITC Life Sciences) set at 4±0.1°C. Rats were lightly restrained and the hind paw was held with the plantar surface on the cold plate. The latency to withdraw the paw was measured to the nearest 0.01secs. To avoid tissue injury, the maximum latency

period permitted was 20 secs. An average of three recordings were taken separated by at least 5 minutes.

RNA extraction and Sequencing

Total RNA isolation and RT-qPCR analysis

Frozen micro dissected spinal cord samples were lysed with Teflon-glass homogenisers and QIAshredder columns (Qiagen). Total RNA was isolated with the Qiagen RNeasy® Mini kit. The RNA was assessed for purity and quantity using the Nanodrop 1000 spectrophotometer and assessed for quality on the Agilent Bioanalyzer 2100. RNA was reverse transcribed with a QuantiTect® Reverse Transcription kit (Qiagen). Levels of BRCA1, TNFα and TNFR1 expression were quantitated in triplicate, relative to GAPDH and triplicate non-template controls for each gene, by RT-qPCR analysis on a LightCycler® 480 instrument with LightCycler® SYBR® Green I Master reagent (Roche). Primer pairs used were; BRCA1 F: 5'-AGGCAAGATCTCGAAGGAACCC-3'; R: 5'-AATCAGGGTCTCTGCTGGAGACTA-3'; TNFα F: 5'-CTGTGCCTCAGCCTCTTCTC-3'; R: 5'-ACTGATGAGAGGGAGCCCAT-3'; TNFR1 F: 5'-CCTCTCTCCCCTCGGCTTTA-3'; R: 5'-CCCGGGTTAGAAAGGCTCAA-3'; GAPDH F: 5'-TGACCTCAACTACATGGTCTACA-3'; R: 5'-GACTCCACGACATACTCAGCA-3'. RT-qPCR cycling parameters were optimised for each gene and were as follows:BRCA1, 400nM Forward and reverse primers, 45 cycles of 95 °C 15 s; 60 °C 40 s; 72 °C 20 s. TNFα and TNFR1, 500nM forward and reverse primers, 45 cycles of 95 °C 10 s; 60 °C 20 s; 72 °C 10 s. Amplicons were confirmed by analysis of melting peaks and agarose gel electrophoresis.

mRNA Library preparation

Four hundred ng of intact high quality total RNA (RIN>7.9) from each sample was then used as input to generate libraries for RNA-sequencing using the NEBNext Ultra II Directional kit (NEB, Cat.no: E7760S) following the manufacturer's recommendations. This protocol involved an initial step of mRNA selection using a poly-A isolation module (NEB, Cat.no: E7490) to select for mRNA with a mature polyA tail, followed by fragmentation prior to first cDNA synthesis and barcoding second-strand cDNA synthesised with indices for Illumina sequencing for final library amplification (10-cycles). The resulting libraries (342-421bp) were assessed on the Bioanalyzer 2100 for purity. The NEBNext Library Quant Kit for Illumina (NEB, Cat.no: E7630L) was used to calculate the quantity of each library. The quantification data was used to pool the libraries in equal molarity prior to performing a QC run on the MiSeq (MiSeq Reagent Kit v3 (150-cycle); Cat no: MS-102-3001). Further deep sequencing was performed on the pooled library over 2 lanes using a HiSeq4000 (by GENEWIZ) to generate roughly 26 million reads per sample.

RNA-Seq Data Analysis

Samples were aligned to the reference genome (Rattus_norvegicus Rnor_6.0.94) with Bowtie2(Langmead and Salzberg, 2012). Aligned sequenced reads per sample were associated to annotated genes with HTSeq (https://htseq.readthedocs.io/en/release_0.11.1/count.html), followed by an inter-sample quantile normalization to correct for technical differences. Differential expression analysis (relative to the samples issued from non-injured and vehicle treated animals) has been performed with Deseq2 (Love et al., 2014). Differentially expressed genes assessed in various conditions (non-injured +C286; Injured +vehicle; Injured +C286) were stratified over three major states (induced, repressed or non-differentially expressed) such that a total of 26 hypothetical combinatorial co-expression events were inferred. Among them,

only 8 appeared as biologically significant situations where the effect of the ligand C286 could be inferred (Figure 1G). To support this hypothesis, all combinatorial co-expression events were analyzed for Gene Ontology (GO) enrichment (DAVID bioinformatics resources),(Huang da et al., 2009) demonstrating relevant enrichment GO terms preferentially in the case of the selected biologically selected co-expression paths.

Microglia cultures

Primary mixed glial cultures were prepared as described previously (Goncalves et al., 2019b). Briefly, mixed glial cultures were obtained from the cortices of C57B/6 postnatal mice (P5–P8). Cultures were maintained at 37 °C (5% CO_2/95% O_2) in medium containing 15% fetal bovine serum (Invitrogen) and 1% penicillin-streptomycin (Sigma Aldrich) for 10–14 days. Microglial cells were then harvested by forceful shaking for 1 min by hand and plated on poly-d-lysine-coated glass coverslips or plastic six-well plates. To obtain reactive microglia, cells were treated with 100ng/ml lipopolysaccharides (LPS) for 3hr (Tarassishin et al., 2014) and then for another 3 hr with either: vehicle; C286 (10^{-7} M); KU55933 (Hickson et al., 2004) (1µM, Abcam, ab120637) or C286 +KU55933.

Immunochemistry and Antibodies

Immunohistochemistry and cytochemistry were carried out as previously described (Goncalves et al., 2005). For the spinal cords: paraffin wax (Pwax) embedded tissues, were first dewaxed in xylene and 100% IMS, then heated in citric acid (10 mM, pH = 6), until boiling, then washed under a running tap for 5 min. For microglia cultures: cells were fixed for 15 min. with 4% PFA. Tissue sections or cells were washed 3x for 5 min each in PBS before incubation with primary antibody in PBS-0.02% Tween at 4ºC overnight. Primary antibody was removed by washing 3x for 5 min each in PBS. They were incubated in the secondary antibody for 1 hr. at

room temperature (RT) in PBS-0.02% Tween, and then washed in PBS 3x for 5 minutes. Antibodies used were: mouse monoclonal anti-βIII tubulin (G7121, Promega, 1:1000); goat polyclonal anti-Iba1 (ab107159, Abcam, 1:2,000); chicken polyclonal anti-Iba1 (ab139590, Abcam, 1:500); rabbit polyclonal anti-FZD10 (ab83044, Abcam, 1:500); rabbit polyclonal anti-BDNF (NBP1-46750, Novus Biologicals,1:1,000); chicken polyclonal anti-GFP (ab13970, Abcam, 1:400) mouse monoclonal anti-TNFα (ab1793, Abcam, 1:500); rabbit polyclonal anti-TNFR1 (ab58436, Abcam, 1:500); rabbit polyclonal anti-NGF 9ab6199, Abcam, 1:100); sheep polyclonal anti-CGRP (BML-CA1137, ENZO, 1;500); rabbit polyclonal anti-BRCA1 (ab191042; Abcam, 1:50); rabbit polyclonal anti-γH2AX (ab2893, Abcam, 1:5,000); mouse monoclonal anti-pATM (sc-47739, Santa Cruz Biotechnology,1:50) ; mouse monoclonal anti-NeuN (MAB377, Millipore, 1:1000); rabbit monoclonal anti-NeuN (#12943, Cell Signaling Technology, 1:3000); rabbit polyclonal anti-P2XR4 (APR-024, alomone labs, 1:20); rabbit polyclonal anti-Daxx (LS-B363**,** LSBio, 1:50). Secondary antibodies were AlexaFluor™ 594, AlexaFluor™ 488 (1:1000, Molecular Probes, Life Technologies) and AlexaFluor™ 647 (1:1000, Molecular Probes, Life Technologies). DAPI was used to stain nuclei (1 μg/mL, Sigma Aldrich).

Immunochemistry quantification

Quantification of protein levels by immunofluorescence (IF) was done as previously described (Herrmann et al., 2010). In brief, positively stained areas were quantified as the pixels of immunoreactivity above a threshold level per unit area using the Zeiss Zen blue edition software. The threshold value was set to include fluorescent positive signal and to exclude background staining. Threshold values for a given section and stain remained the same throughout the study and the quantifications were done by an operator blinded to the treatments.

Confocal microscopy

Multichannel fluorescence (DAPI–FITC–Texas Red filter set) images were captured using a Zeiss LSM 700 laser-scanning confocal microscope. For high magnification images, a 63 x oil-immersion Aprochromat objective (Carl Zeiss) was used. Settings for gain, aperture, contrast and brightness were optimized initially, and held constant throughout each study so that all sections were digitized under the same conditions of illumination. Channels were imaged sequentially to eliminate bleed-through, and multichannel image overlays were obtained using Adobe Photoshop 7.0 (Adobe Systems).

Western blotting

Spinal cord proteins were isolated from the phenol fraction remaining after QIAzol RNA extraction, according to the Qiagen user-developed protocol RY16. Briefly, the phenolic phase was treated with ethanol and the proteins precipitated by isopropanol. Protein pellets were sequentially washed with 0.3M guanidine-hydrochloride in 95% ethanol and 100% ethanol, air dried, then resuspended in 10M urea, 50mM DTT in water. Protein concentrations were measured by Coomassie-Bradford assays and proteins were separated by SDS-PAGE from 20µg aliquots loaded on to 12% (w/v) Bis-Tris polyacrylamide gels. For BDNF analysis the separated proteins were blotted to a 0.45µm pore size nitrocellulose membrane (BA85; Schleicher and Schuell) with a Trans-Blot SD Semi-Dry Transfer Cell (Bio-Rad Laboratories). The membrane was incubated in Odyssey® Blocking Buffer (PBS) (LICOR Bioscience) for 1 h at RT followed by an overnight incubation at 4°C with the primary antibody in blocking buffer. Anti-β-actin antibody was then added, for 1 h at room temperature (RT), after which the membrane was washed with PBS containing 0.1%v/v Tween-20 and incubated for a further 1 h at RT with secondary antibodies in blocking buffer. Finally, the membrane was washed as before and simultaneously scanned at 700nm and 800nm using an Odyssey® protein detection

system (LICOR Bioscience). Proteins were quantified using Image Studio Lite software (LICOR Bioscience) and normalized against β-actin. For the detection of BRCA1, proteins were transferred to a Hybond™-ECL membrane (GE Healthcare). The membrane was cut at the 76kD marker and both sections incubated in a blocking buffer consisting of 1% (w/v) BSA in TBS-Tween-20 (0.1%, v/v) for 1 h at RT followed by an overnight incubation at 4°C with anti-BDNF antibody added to the top section only. The top membrane was washed with TBS-Tween-20 (0.1% v/v) then incubated with a biotinylated secondary antibody for 1 h at RT, washed as before and incubated with ECL™ Western Blotting Detection Reagents (GE Healthcare). Proteins were visualised with the BioSpectrum Imaging system (UVP). The lower section of the membrane was incubated with a β-actin antibody for 1 h at RT followed by washing with TBS-Tween-20 (0.1% v/v) and incubation for a further at 1 h at RT with secondary antibodies in blocking buffer. β-Actin was visualised with the Odyssey® protein detection system (LICOR Bioscience) as above. Antibodies: Rabbit polyclonal anti-BRCA1 (ab191042, Abcam, 1:500); mouse monoclonal anti-BDNF (Ab205067, Abcam, 1:500); Rabbit polyclonal anti-β-actin (Ab8227, Abcam, 1:5000); Biotinylated Goat Anti-Rabbit IgG (BA-1000, Vector Laboratories, 1:500); Alexa Fluor680 (1:5000, Invitrogen) and IR Dye 800CW (1:5000, LICOR Biosciences).

Statistical analysis

Data and statistical analysis comply with the recommendations on experimental design and analysis in pharmacology (Curtis and Abernethy, 2015). Data analysis was performed in a blinded fashion. All statistical analysis was performed using Sigma Stat Software (SPSS Software Ltd, Birmingham, UK) using unpaired *t*-tests and one-way ANOVA with Pairwise Multiple Comparison Procedures (Tukey Test), or two-way ANOVA with Pairwise Multiple Comparison Procedures (Holm-Sidak method) as indicated in the Figure legends. *$p \leq 0.05$,

**$p \leq 0.01$, ***$p \leq 0.001$. Exact p values are shown when p> 0.001. *n* represents the number of biological replicates. Experiments were repeated to ensure reproducibility of the observations. No statistical methods were used to predetermine sample size.

Data and Software Availability

Raw and processed transcriptome datasets are available under the GEO accession number (GSE135080) and the Mendeley repository Mendeley Data, v1 **http://dx.doi.org/10.17632/kjvs5vgkbf.1**DOI .

Supplemental references

Bennett, G.J., Chung, J.M., Honore, M., and Seltzer, Z. (2003). Models of neuropathic pain in the rat. Curr Protoc Neurosci *Chapter 9*, Unit 9 14.

Curtis, M.J., and Abernethy, D.R. (2015). Revision of instructions to authors for pharmacology research and perspectives: enhancing the quality and transparency of published work. Pharmacol Res Perspect *3*, e00106.

Goncalves, M.B., Boyle, J., Webber, D.J., Hall, S., Minger, S.L., and Corcoran, J.P. (2005). Timing of the retinoid-signalling pathway determines the expression of neuronal markers in neural progenitor cells. DevBiol *278*, 60-70.

Goncalves, M.B., Clarke, E., Jarvis, C.I., Barret Kalindjian, S., Pitcher, T., Grist, J., Hobbs, C., Carlstedt, T., Jack, J., Brown, J.T.*, et al.* (2019a). Discovery and lead optimisation of a potent, selective and orally bioavailable RARbeta agonist for the potential treatment of nerve injury. Bioorg Med Chem Lett *29*, 995-1000.

Goncalves, M.B., Wu, Y., Clarke, E., Grist, J., Hobbs, C., Trigo, D., Jack, J., and Corcoran, J.P.T. (2019b). Regulation of myelination by exosome associated retinoic acid release from NG2-positive cells. J Neurosci.

Herrmann, J.E., Shah, R.R., Chan, A.F., and Zheng, B. (2010). EphA4 deficient mice maintain astroglial-fibrotic scar formation after spinal cord injury. Exp Neurol *223*, 582-598.

Hickson, I., Zhao, Y., Richardson, C.J., Green, S.J., Martin, N.M., Orr, A.I., Reaper, P.M., Jackson, S.P., Curtin, N.J., and Smith, G.C. (2004). Identification and characterization of a novel and specific inhibitor of the ataxia-telangiectasia mutated kinase ATM. Cancer Res *64*, 9152-9159.

Huang da, W., Sherman, B.T., and Lempicki, R.A. (2009). Bioinformatics enrichment tools: paths toward the comprehensive functional analysis of large gene lists. Nucleic Acids Res *37*, 1-13.

Kilkenny, C., Browne, W., Cuthill, I.C., Emerson, M., Altman, D.G., and Group, N.C.R.R.G.W. (2010). Animal research: reporting in vivo experiments: the ARRIVE guidelines. J Gene Med *12*, 561-563.

Langmead, B., and Salzberg, S.L. (2012). Fast gapped-read alignment with Bowtie 2. Nat Methods *9*, 357-359.

Love, M.I., Huber, W., and Anders, S. (2014). Moderated estimation of fold change and dispersion for RNA-seq data with DESeq2. Genome Biol *15*, 550.

McGrath, J.C., and Lilley, E. (2015). Implementing guidelines on reporting research using animals (ARRIVE etc.): new requirements for publication in BJP. Br J Pharmacol *172*, 3189-3193.

Tarassishin, L., Suh, H.S., and Lee, S.C. (2014). LPS and IL-1 differentially activate mouse and human astrocytes: role of CD14. Glia *62*, 999-1013.
